# Rapid Screening of Essential Oils as Substances Which Enhance Antibiotic Activity Using a Modified Well Diffusion Method

**DOI:** 10.3390/antibiotics10040463

**Published:** 2021-04-20

**Authors:** Ze-Hua Cui, Hui-Ling He, Shuai-Bin Wu, Chun-Liu Dong, Si-Ya Lu, Ti-Jiang Shan, Liang-Xing Fang, Xiao-Ping Liao, Ya-Hong Liu, Jian Sun

**Affiliations:** 1 National Risk Assessment Laboratory for Antimicrobial Resistance of Animal Original Bacteria, South China Agricultural University, Guangzhou 510642, China; cuizehua@stu.scau.edu.cn (Z.-H.C.); hhl@stu.scau.edu.cn (H.-L.H.); 1074298292@stu.scau.edu.cn (S.-B.W.); lsy@stu.scau.edu.cn (S.-Y.L.); fanglx@scau.edu.cn (L.-X.F.); xpliao@scau.edu.cn (X.-P.L.); lyh@scau.edu.cn (Y.-H.L.); 2 Guangdong Provincial Key Laboratory of Veterinary Pharmaceutics Development and Safety Evaluation, South China Agricultural University, Guangzhou 510642, China; 3 College of Veterinary Medicine, Northeast Agricultural University, Harbin 150030, China; dcliu@neau.edu.cn; 4 College of Forestry and Landscape Architecture, South China Agricultural University, Guangzhou 510642, China; tjshan@scau.edu.cn; 5 Guangdong Laboratory for Lingnan Modern Agriculture, Guangzhou 510642, China

**Keywords:** essential oils, antibiotic adjuvant, modified well diffusion method, combination therapy

## Abstract

Antimicrobial resistance is recognized as one of the major global health challenges of the 21st century. Synergistic combinations for antimicrobial therapies can be a good strategy for the treatment of multidrug resistant infections. We examined the ability of a group of 29 plant essential oils as substances which enhance the antibiotic activity. We used a modified well diffusion method to establish a high-throughput screening method for easy and rapid identification of high-level enhancement combinations against bacteria. We found that 25 essential oils possessed antibacterial activity against *Escherichia Coli* ATCC 25922 and methicillin-resistant *Staphylococcus aureus* (MRSA) 43300 with MICs that ranged from 0.01% to 2.5% v/v. We examined 319 (11 × 29) combinations in a checkerboard assay with *E. Coli* ATCC 25922 and MRSA 43300, and the result showed that high-level enhancement combinations were 48 and 44, low-level enhancement combinations were 214 and 211, and no effects combinations were 57 and 64, respectively. For further verification we randomly chose six combinations that included orange and Petitgrain essential oils in a standard time-killing assay. The results are in great agreement with those of the well diffusion assays. Therefore, the modified diffusion method was a rapid and effective method to screen high-level enhancement combinations of antibiotics and essential oils.

## 1. Introduction

Bacterial resistance to antibiotics is primarily spread by horizontal transfer of drug resistance genes or through mutation [1]. Clinical bacterial isolates are commonly multiple drug resistant (MDR) and these have increased in the past decade [2]. The latter is especially concerning for the human pathogenic bacteria in the ESKAPE group; *Enterococcus faecium*, *Staphylococcus aureus*, *Klebsiella pneumoniae*, *Acinetobacter baumannii*, *Pseudomonas aeruginosa*, and *Enterobacter*. Infections by these pathogens can result in treatment failure, increased mortality, as well as high therapy costs [3]. However, treatment options are becoming limited and new treatment strategies or therapeutic agents are needed to combat MDR bacteria. One option for improving antibiotic effectiveness is combination therapy [4]. The use of plant essential oils as an antimicrobial adjuvant has been successful in improving the effectiveness of drugs already in clinical use [5,6].

Essential oils or volatile oils are produced by secondary metabolism in a group of aromatic plants and display a variety of biological properties and possess antimicrobial, antiviral, anti-inflammatory, and antioxidant activities [7]. These oils are complex mixtures that consist of 20–60 components at different concentrations. In general, 2–3 components predominate and represent 20–70% of the total [8]. Additionally, the use of essential oils as antimicrobial compounds has advantages including minimal side effects, good tolerance, biodegradability, and low cost [9]. In addition, essential oils are safe and can be used as substances which enhance antibiotic activity [10,11]. Numerous studies have demonstrated that essential oils administered with conventional antibiotics can increase the effectiveness and reverse bacterial resistance and clear MDR bacteria from infections. Coriander essential oil showed synergy with antibiotics against methicillin-resistant *Staphylococcus aureus* (MRSA) and other Gram-positive bacteria (methicillin-susceptible *S. aureus*, *S. epidermidis*), but also Gram-negative bacteria (*P. aeruginosa*, *E. coli*) [12].

Currently, there is no standardized method to screen the antibacterial activities of essential oils. The methods in current use are the disk diffusion test, agar dilution test, well diffusion test, and broth microdilution [13]. The checkerboard method and time-kill tests are also recommended to detecting interactive effects among antibiotics. These methods are important but time-consuming, and the number of tested drug combinations can be limited [14]. In addition, due to the different test assays, inoculum size, growth media, culture conditions, and cut-off determinations, it is difficult to compare between studies. In the current study, we established a standard well diffusion method for rapid screening of combinations of essential oils and antibiotics, and time-kill assay was performed to verify some combinations and screen out effective antimicrobial substances which enhance antibiotic activity.

## 2. Results

### 2.1. MIC Values of Essential Oils and Antimicrobial Agents

We initially examined the activity of a collection of essential oils for their antibacterial activity against E. coli ATCC 25922. We found strong antibacterial activity for compounds 2 (lemongrass), 12 (sweet scented geranium), 19 (Chinese cinnamon), 20 (cinnamon leaf), 25 (true cinnamon tree), and 27 (caraway) with MIC values that ranged from 0.01% to 0.078% for E. coli ATCC 25922. In contrast, essential oils derived from turmeric (16), Indian frankincense (22), carrot seeds (23), and anise (26) were not biologically active at the maximum concentration (MIC values > 5%). Additionally, the antibacterial activity of essential oils against MRSA 43300 was also examined to discover the effects of essential oils on Gram-positive bacteria. We found strong antibacterial activity for compounds 2 (lemongrass), 6 (orange), 10 (abies alba), 12 (sweet scented geranium), 13 (tea tree), 19 (Chinese cinnamon), 20 (cinnamon leaf), 24 (cinnamon bark), and 27 (caraway) with MIC values that ranged from 0.01% to 0.078%, and compound 11 (fennel seed) and compound 18 (grapefruit pink) were not biologically active at the maximum concentration with MIC values > 5%. The other essential oils showed different antibacterial activities against the tested bacteria, with MIC values ranging from 0.15 to 2.5% (Table 1). The validity of these MIC tests was confirmed with the quality control standards based on CLSI guidelines (Table 2).

**Figure A1:**
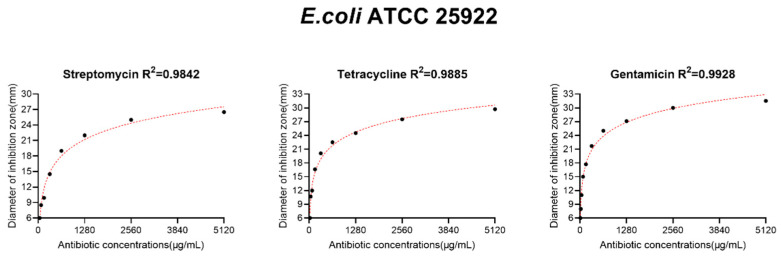
The optimum concentration of antibiotics against *E. coli* ATCC 25922 (partial).

**Figure A2:**
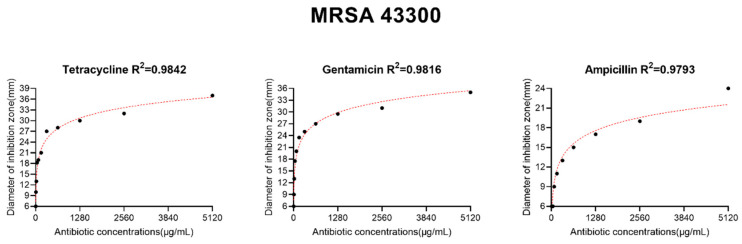
The optimum concentration of antibiotics against MRSA 43300 (partial).

The levels of the oils that maximized the antibacterial effects were determined using agar well diffusion (Figure A1 and Figure A2). The optimal concentrations of essential oils ranged from 100% to 0.39% (Table 1). The antimicrobials were optimal when added in a range of 5120 to 20 μg/mL and produced inhibition zones between 11 and 18 mm with the exception of vancomycin and bacitracin for E. coli ATCC 25922 and erythromycin for MRSA 43300 (Table 2).

### 2.2. Effects of Essential Oils and Antibiotic Combinations

**Figure 1:**
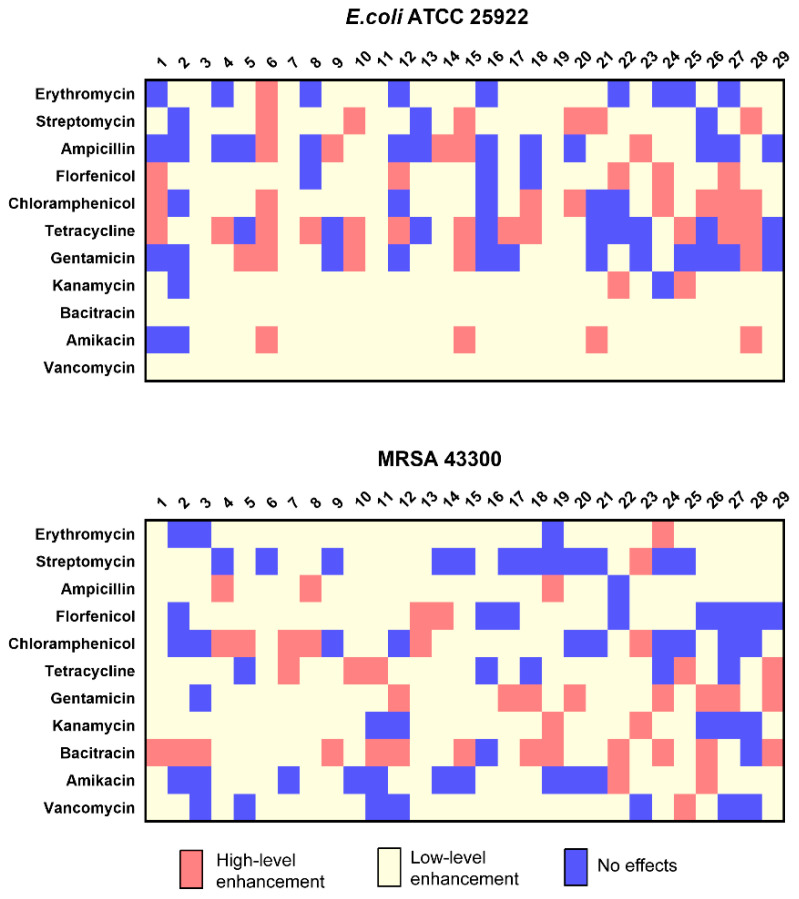
Inhibition zone diameter alterations using antibiotic and essential oil combinations. Red, high-level enhancement; yellow, low-level enhancement; blue, no effects.

The optimal levels for these compounds were then tested in combinations of essential oils and antibiotic using a modified well diffusion method. We examined 319 (11 × 29) combinations and in 48/319 the inhibition zone diameters were increased >2 mm over the single drug alone. Additionally, 214/319 increased from 0–2 mm and in 57/319 the zones of inhibition were reduced. These results indicated that there were 48 high-level enhancement combinations and 47 combinations resulted in inhibition zone diameter increases of 2.5 mm with exception of Petitgrain combined with tetracycline. In the group of 29 essential oils, orange (6), Petitgrain (15), and bergamot (28) were high-level enhancement with 6, 8, and 10 antibiotics, respectively. The same test was performed with MRSA 43300, and we found 44/319 the inhibition zone diameters were increased >2 mm, 211/319 increased from 0–2 mm, and in 64/319 the zones of inhibition were reduced (Figure 1). The results showed 44 high-level enhancement combinations were found, and the corresponding inhibition zone diameter of these combinations increased by 3 mm or more (Figure 1).

### 2.3. In Vitro Time-Killing Curves

**Figure 2:**
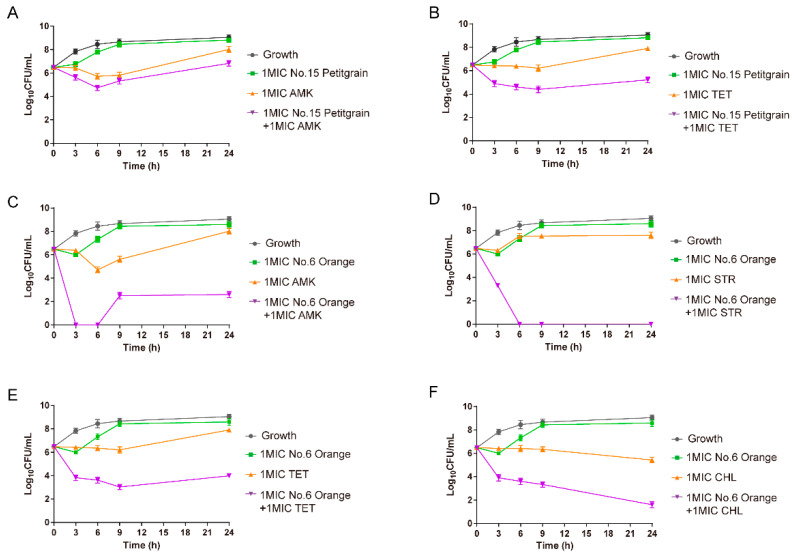
Time-kill curves showing effects of Petitgrain (15) with (**A**) amikacin (**B**) tetracycline and orange (6) with (**C**) amikacin, (**D**) streptomycin, (**E**) tetracycline, and (**F**) chloramphenicol both at 1× MIC against *E. coli* ATCC 25922.

To verify whether the high-level enhancement combinations screened by the modified well diffusion method were effective, we randomly selected 6 high-level enhancement combinations for further verification using a time-killing assay. These combinations include easily available and low-cost essential oils like Petitgrain (15) and Orange (6), as well as clinically common antibiotics like amikacin, tetracycline, and chloramphenicol. We found that the combination of Petitgrain oil and amikacin or tetracycline at 1× MIC caused 1.19- and 2.68-log_10_ CFU/mL reductions as compared with the most active antimicrobial alone at 24 h, respectively. This demonstrated additive and synergistic effects, respectively. The combinations of orange oil (6) and amikacin, streptomycin, tetracycline, and chloramphenicol displayed rapid decreases in bacterial numbers than with individual drugs. The reductions were 5.44-, 7.60-, 3.94-, and 3.85-log_10_ CFU/mL as compared with the most active antimicrobial alone at 24 h, respectively (Figure 2). These data indicated that the modified well diffusion method could serve as an effective screen to examine high-level enhancement combinations of antibiotics and essential oils.

**Figure 3:**
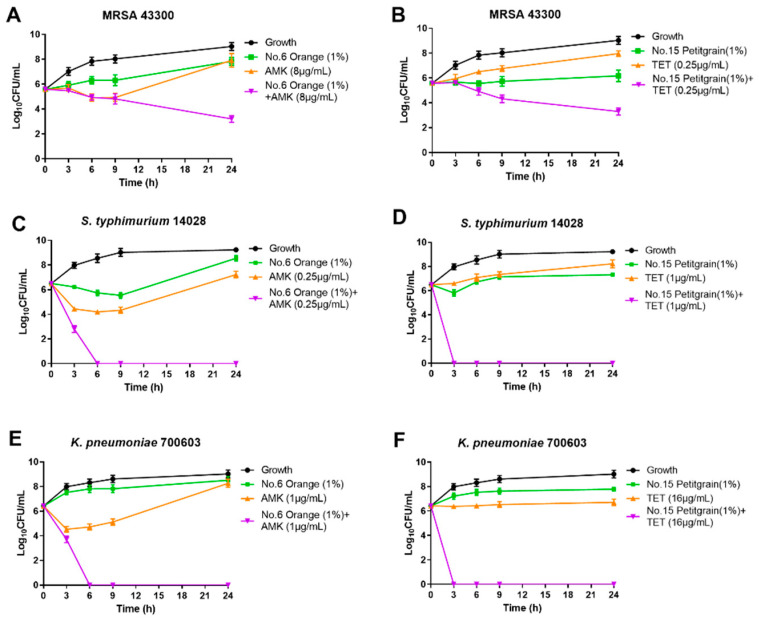
Time-kill curves of combinations of orange (1%) and amikacin (1× MIC) against (**A**) MRSA 43300 (**C**) *S. typhimurium* ATCC 14028 and (**E**) *K. pneumoniae* ATCC 700603 and the combinations of Petitgrain (1%) and tetracycline (1× MIC) against (**B**) MRSA 43300 (**D**) *S. typhimurium* ATCC 14028 and (**F**) *K. pneumoniae* ATCC 700603.

Three other bacterial strains also showed significant synergistic effects with amikacin and orange oil (6). This combination completely inhibited bacterial growth at 6 h against *Salmonella. typhimurium* and *K. pneumoniae* and caused a 4.63-log_10_ decrease at 24 h for the MRSA strain (Figure 2). The combination of tetracycline and Petitgrain oil (15) completely inhibited bacterial growth at 3 h against the Gram-negative bacteria *S. typhimurium* and *K. pneumoniae* and caused a 2.25-log_10_ decrease at 24 h for the Gram-positive bacterium MRSA (Figure 3).

## 3. Discussion

The rapid emergence of MDR bacteria poses a huge threat to global public health since these dramatically decrease clinical treatment options. Combination therapies are commonly used in the clinical setting against MDR infections and many have proven to be effective [15,16]. In particularly, promising substances which enhance antibiotic activity are essential oils [17].

We examined 29 essential oils in this study and those derived from the cinnamon plants *C. cassia* and *C. zeylanicum* possessed the most robust antibacterial activity against *E. coli* with MIC values <0.01%. The primary volatile oil present in these preparations is cinnamaldehyde [18]. The *C. citratus* and *P. graveolens* essential oils also possessed good antibacterial activities and the primary components of these are citral and citronellol, respectively [19,20]. The orange and Petitgrain oils possessed significant synergistic effects with many of the antibiotics we used for this study even though their antibacterial properties when used alone were not significant.

Orange and Petitgrain are both *Citrus aurantium* derivatives and are generally recognized as safe for used in foods. Citrus oils have been successfully used as antimicrobials against *S. aureus*, *E. coli*, *S. typhimurium* and *K. pneumoniae* [21,22,23]. The primary components of orange oil are linalool and decanal and these demonstrated good inhibition of *S. aureus* growth [24]. Essential oils may alter drug permeability and allow antibiotic permeation of the cell [25]. Similarly, some components of essential oils may directly damage the membrane structure, which could lead to intracellular material leakage and enhance the action of antibiotics [6].

A variety of laboratory methods can be used to screen synergistic combinations between essential oils and antibiotics including agar disk diffusion, checkerboard and time-kill assays [26]. A recent study demonstrated that the combination of *Thymus vulgaris* essential oil and cefotaxime was synergistic against blaSHV-12 producing *E. coli* (FICI 0.28) using the checkboard method [11]. As regards *Thymus vulgaris* essential oil, a recent study found synergistic combinations among essential oil compounds of thyme oil, using the checkerboard assay [27]. Therefore, future works could be addressed to find synergistic combinations among essential oil compounds and antibiotics. Another investigation using disk diffusion demonstrated that basil, clary sage, and rosemary essential oils combined with antibiotics were synergistic antibacterial combinations [28]. The latter method is employed most frequently when evaluating antibacterial activity or screening synergistic combinations [29]. The disc thickness, soaking time, and the volume of test substance needed are all disadvantages for disc diffusion. The use of the well diffusion method to evaluate the antibacterial activity of natural products against *S. aureus* and *E. coli* has demonstrated better sensitivity than the disc diffusion method [30]. Another study also demonstrated a greater sensitivity for well diffusion over disc diffusion using Allium essential oils against a group of microbial pathogens [31].

In this work, we modified the well diffusion method and established a high-throughput screening method for easy and rapid identification of synergistic combination of antibiotics and essential oils against bacteria. At present, different laboratories use different screening methods for these types of investigations so it is difficult to compare them. The use of a common method for selecting essential oils is conducive to data sharing between researchers and the modified well diffusion method is a good way to do this. We were able to rapidly test 11 antimicrobial and 29 essential oil combinations and found that orange essential oil increased the inhibition zones for amikacin, streptomycin, tetracycline, and chloramphenicol as well as for Petitgrain oil and tetracycline. In addition, we confirmed these results using the standard time-kill assay and the two results were highly consistent. The well diffusion method has advantages such as a high-throughput format that can quantify results with many combinations and bacterial isolates. Previous studies have used the well method to evaluate the antibacterial activity of lemongrass and eucalyptus essential oils against four bacterial pathogens [32]. The well diffusion method provided a fast, cost effective, low tech and generally reliable method for large-scale screening of essential oils as substances which enhance antibiotic activity.

Importantly, to the best of our knowledge, this is the first study demonstrating high-level enhancement effects between orange essential oil with amikacin, streptomycin, tetracycline, and chloramphenicol and Petitgrain essential oil and tetracycline against *E. coli.* Interestingly, the combinations orange oil/amikacin and Petitgrain oil/tetracycline were also synergistic against MRSA, *S. typhimurium* and *K. pneumoniae* using the time-kill assay. The mechanism of action for these combinations is not yet clear but may include increased membrane permeability, reduced membrane potential, alterations of proton and ion channels, a disruption of protein metabolism, and disruption of cellular components [33].

## 4. Materials and Methods

### 4.1. Antimicrobials and Essential Oils

Bacitracin (BCR), ampicillin (AMP), gentamicin (GEN), kanamycin (KAN), tetracycline (TET), erythromycin (ERY), chloramphenicol (CHL), florfenicol (FFC), streptomycin (STR), amikacin (AMK), and vancomycin (VAN) were selected for our studies based on their mechanisms of action. All study antimicrobial agents were purchased from Guangzhou Xiang Bo Biological Technology Co. Ltd. (Guangzhou, China). Antibiotic stocks solutions (5120 μg/mL) were prepared with suitable solvents according to the manufacturer’s recommendations. Twenty-nine different essential oils used in this study were both purchased from FRANCINE CHICARD Co. Ltd. (Guangzhou, China) (Table 1). All essential oils were directly used in the original solution (100%) and diluted with 1% dimethylsulfoxide in the experiment.

### 4.2. Bacterial Strains and Growth Conditions

*E. coli* ATCC 25922, *S. typhimurium* ATCC 14028, *K. pneumoniae* ATCC 700603 and methicillin –resistant *S. aureus* ATCC 43300 (MRSA) were used in this study. The bacterial strains except MRSA were grown on MacConkey agar (MHA; Oxoid, Cambridge, UK), MRSA were grown on Manitol salt agar (MSA; Oxoid, Cambridge, UK). Single colonies were selected and incubated in Mueller-Hinton broth (MHB, Oxoid) at 37 °C with shaking at 200 rpm until an optical density equal to 0.5 McFarland units was reached.

### 4.3. Determination of Minimum Inhibitory Concentration (MIC)

The MICs of antimicrobials and essential oils were conducted in triplicate by broth microdilution method as recommended by the Clinical and Laboratory Standards Institute guidelines (CLSI, 2018). Simultaneously, the MICs of essential oils were determined using MHB supplemented agar (0.15% w/v). The essential oils were two-fold serially diluted from 5% to 0.0098% (v/v) in a 96-well microplate in 100 μL volumes. Then 100 μL of each bacterial suspension in MHB was added to microplates so that the final cellular concentration was 5 × 10^5^ CFU/mL. Plates were incubated at 37 °C for 16 h. At this time, 10 μL resazurin aqueous solutions (0.1 mg/mL) were added to each well and the plates were incubated for 2 h at 37 °C. The MIC was then determined as the lowest concentration that prevented the blue color change into pink [34].

### 4.4. Screening for Synergistic Effects of Essential Oils with Antibiotics Using Modified Well Diffusion

Total of 20 mL tempered MH agar medium was poured into 90 mm bacteriological Petri dishes. Samples of 100 μL overnight cultures (0.5 McFarland) of *E. coli* ATCC 25922 were spread onto the agar plate surface. After hardening, 7 mm × 6 mm holes were made in the plates and its bottom was sealed. Then, a drop of heated liquid MH agar was added to the bottom of the hole to ensure that antibiotics or essential oils do not flow from the bottom. Finally, the test compounds were applied [35]. The same protocol was performed with MRSA 43300.

The essential oils and antibiotics were two-fold serially diluted ranging from 100% to 0.39% and 5120 to 20 μg/mL, respectively. The test compounds were added to the well in 10 μL volumes and to test for synergistic effects, antimicrobials and essential oils were added together at the same volume. The plates were then incubated at 37 °C for 18 h. The inhibition zone diameter (mm) was measured using a Vernier caliper. The optimum concentration of antibiotics is defined as a concentration that produces the inhibition zone diameter 12 to 17 mm, except for antibiotics that have no antibacterial activity against *E. coli* or *S. aureus*, such as vancomycin and bacitracin. The optimum concentration of essential oils is defined as the first concentration that does not produce the inhibition zone. All tests were performed in triplicate. There was a linear relationship between the diameter of the inhibition zone and the antibiotic concentrations in a certain range when the antibacterial activity of antibiotics was evaluated by well diffusion method [36]. Among the antibiotics involved in this study, when the inhibition zone diameter is in the range of 12–17 mm, the antibiotic concentration usually needs to increase by at least 50% for every 2 mm increase in the inhibition zone diameter, so 2 mm is used as the cut-off value. The following criteria were used to evaluate interaction of antibiotics and essential oil: compared with antibiotic alone, diameter of combination group inhibition zone in the increased >2 mm high-level enhancement; 0–2 mm low-level enhancement; <0 no effects.

### 4.5. Time-Kill Assays

Time-kill experiments were conducted to further characterize the synergistic activity of the essential oil and antibiotic combinations as previously described [37]. In brief, an initial inoculum of ~10^6^ CFU/mL logarithmic phase cells incubated with antibiotics (1× MIC) in the presence and absence of essential oils (1× MIC) and time–kill curves were compared to assess efficacy. Serial samples were obtained at 0, 3, 6, 9, and 24 h after incubation at 37 °C. Bacterial counts were determined based on the quantitative cultures on MHA plates. Synergy was defined as achieving a ≥2 log_10_ CFU/mL reduction in bacterial growth at 24 h with the combination compared with the most active individual drug concentration used on its own [26]. Three independent experimental runs were performed.

### 4.6. Statistical Analysis

Bacterial counts were transformed to log values and the data were analyzed using Graphpad Prism 7.0 (GraphPad Software, San Diego, CA, USA). One-way analysis of variance and Student’s t-test were used for the analysis. A *p*-value of ≤0.05 was considered significant.

## 5. Conclusions

In conclusion, we modified the well diffusion method and established a standardized condition for rapid screening of high-level enhancement combinations of essential oils and convention antimicrobials. This study identified novel synergistic combinations of orange essential oil and amikacin, streptomycin, tetracycline, and chloramphenicol as well as Petitgrain essential oil and tetracycline against *E. coli*. Moreover, the combination of orange oil/amikacin and Petitgrain oil/tetracycline had synergistic activity against *S. aureus*, *S. typhimurium*, and *K. pneumoniae*. Essential oils from citrus alone or in combination with antibiotics could potentially act as broad-spectrum antibiotics against both Gram-positive and Gram-negative bacteria. Future studies should confirm the reproducibility of these results in larger collections of clinical strains and define the molecular mechanisms of synergistic activities of essential oils (e.g., thymol, cinnamaldehyde, citral, carvacrol).

## Figures and Tables

**Table 1:** Origin of essential oils and MIC values and optimum concentrations against *E. coli* ATCC 25922 and MRSA 43300.

Number	Essential Oils	Origin	MIC (*v/v*%)		Optimum Concentrations (*v/v*%)	
			*E. coli*	*S. aureus*	*E. coli*	*S. aureus*
1	Holly	Inadia	2.500	5.000	100	100
2	Cymbopogon citratus	India	0.078	0.039	3.13	12.50
3	Verbenone	France	0.313	0.313	12.50	25
4	Tangerine leaf	Italy	0.313	2.500	100	100
5	Tangerine	Italy	0.625	1.250	12.50	12.50
6	Orange	Italy	1.250	0.039	25	50
7	Lime oil	India	0.313	0.625	6.25	12.50
8	Lemon	Italy	0.313	0.156	6.25	50
9	Dill	Austria	0.313	0.625	100	6.25
10	Abies alba	Austria	0.156	0.078	3.13	6.25
11	Fennel	France	2.500	>5	100	100
12	Pelargonium graveolens	Egypt	0.039	0.039	25	50
13	Tea tree	India	0.156	0.078	6.25	6.25
14	Abies sibirica	Austria	0.156	0.156	6.25	25
15	Petitgrain	France	0.625	1.250	100	50
16	Curcuma longa	India	>5	2.500	100	100
17	Lavandula spica	France	0.156	0.625	12.50	6.25
18	Citrus paradisi	Italy	0.625	>5	6.25	100
19	Cinnamomum cassia	India	<0.01	0.019	0.78	0.39
20	Cinnamon Leaf	Sri Lanka	0.019	0.039	0.78	6.25
21	Eucalyptus globulus	Australia	0.313	0.313	12.50	1.56
22	Boswellia serrata	India	>5	2.500	50	100
23	Carrot Seed	France	>5	0.313	100	100
24	Cinnamomum zeylanicum	Sri Lanka	<0.01	0.039	0.78	6.25
25	Piper nigrum	India	2.500	1.250	100	12.50
26	Pimpinella anisum	India	>5	5.000	100	100
27	Trachyspermum ammi	India	0.019	0.019	0.78	12.50
28	Bergamot	India	2.500	1.250	50	6.25
29	Cupressu	France	0.313	1.250	6.25	25

**Table 2:** MIC values and optimum concentrations of conventional antibiotics against *E. coli* ATCC 25922 and MRSA 43300. (Inhibition zones refers to optimum concentrations).

Antibiotics	Classification	MIC (μg/mL)		Optimum Concentrations (μg/mL)		Inhibition Zones (mm)	
		*E. coli*	*S. aureus*	*E. coli*	*S. aureus*	*E. coli*	*S. aureus*
Ampicillin	Beta-lactam antibiotic	2	1	1280	640	11	15
Kanamycin	Aminoglycosides	4	64	320	5120	14	10
Erythromycin	Macrolides	4	>256	2560	5120	6	6
Chloramphenicol	Amphenicols	4	2	320	320	14	18
Florfenicol	Amphenicols	1	1	2560	160	17	16
Streptomycin	Aminoglycosides	8	2	320	320	14.5	14
Amikacin	Aminoglycosides	1	0.5	160	640	15	13
Gentamicin	Aminoglycosides	0.5	0.25	160	20	16	13
Tetracycline	Tetracyclines	1	0.125	160	20	14	13
Bacitracin	Polypeptide	>256	64	5120	2560	6	14.5
Vancomycin	Glycopeptides	>256	1	5120	160	6	15

## Data Availability

Not applicable.

## Appendix A

In this study, the optimum concentration of antibiotics is defined as a concentration that produces the inhibition zone diameter 12 to 17 mm, except for antibiotics with no antibacterial activity. The optimum concentration was determined by the modified well diffusion method, and the results showed most of the antibiotics used in the experiment have different degrees of antibacterial activity against bacteria *E. coli* ATCC 25922 and MRSA 43300 (Figure A1 and Figure A2).
